# Feedforward-Feedback Hybrid Control for Magnetic Shape Memory Alloy Actuators Based on the Krasnosel'skii-Pokrovskii Model

**DOI:** 10.1371/journal.pone.0097086

**Published:** 2014-05-14

**Authors:** Miaolei Zhou, Qi Zhang, Jingyuan Wang

**Affiliations:** College of Communication Engineering, Jilin University, Changchun, China; Washington State University, United States of America

## Abstract

As a new type of smart material, magnetic shape memory alloy has the advantages of a fast response frequency and outstanding strain capability in the field of microdrive and microposition actuators. The hysteresis nonlinearity in magnetic shape memory alloy actuators, however, limits system performance and further application. Here we propose a feedforward-feedback hybrid control method to improve control precision and mitigate the effects of the hysteresis nonlinearity of magnetic shape memory alloy actuators. First, hysteresis nonlinearity compensation for the magnetic shape memory alloy actuator is implemented by establishing a feedforward controller which is an inverse hysteresis model based on Krasnosel'skii-Pokrovskii operator. Secondly, the paper employs the classical Proportion Integration Differentiation feedback control with feedforward control to comprise the hybrid control system, and for further enhancing the adaptive performance of the system and improving the control accuracy, the Radial Basis Function neural network self-tuning Proportion Integration Differentiation feedback control replaces the classical Proportion Integration Differentiation feedback control. Utilizing self-learning ability of the Radial Basis Function neural network obtains Jacobian information of magnetic shape memory alloy actuator for the on-line adjustment of parameters in Proportion Integration Differentiation controller. Finally, simulation results show that the hybrid control method proposed in this paper can greatly improve the control precision of magnetic shape memory alloy actuator and the maximum tracking error is reduced from 1.1% in the open-loop system to 0.43% in the hybrid control system.

## Introduction

Development of new smart materials like piezoelectric, magnetostrictive and shape memory alloy (SMA) made it possible to produce micro motion and force, and magnetic shape memory alloy (MSMA) is also a new type of material which possesses advantages of smart materials such as fast response frequency, outstanding strain and stress capability [1]–[6]. The MSMA material changes shape when exposed to a large magnetic field and the shape variable is up to 10–15%. MSMA has been successfully actuated at frequencies well above 1 kHz at present [7]–[11]. Therefore, MSMA material has great potential for development in the micro drive and micro positioning actuator manufacturing field and it leads to simple, light and reliable system construction. However, there exists nonlinear hysteresis phenomenon between input control signal and output displacement in practical application of the MSMA actuator which reduces the control accuracy of the system, makes the system oscillate and even leads to the instability of the system [11]–[16].

Many researchers have proposed a number of control strategies to eliminate the negative effects of hysteresis nonlinearity. Tao G et al. proposed a parameterized hysteresis model, developed a hysteresis inverse, and designed adaptive controllers based on an adaptive inverse model, which improved system performance using a robust adaptive law to update the controller parameters and hysteresis inverse parameters [16]. Zhou ML et al. proposed a hybrid control method comprising a feedforward loop with the inverse Prandtl-Ishlinskii (PI) model and a feedback loop with a neural network controller, and simulation and experimental results showed that the maximum error rate of the hybrid controller based on the inverse PI model was 1.37% [17]. Ge P et al. proposed a hysteresis control approach for the piezoceramic actuator that incorporated a feedforward loop using the classical Preisach model with a Proportion Integration Differentiation (PID) feedback controller, which improved control accuracy by 50% compared to a regular PID controller [18]. Sui XM et al. proposed a complex control strategy by combining a Cerebellar Model Articulation Controller neural network feedforward control, which was used to establish a real-time hysteresis model for Giant Magnetostrictive Material and a sliding mode variable structure control that was used to eliminate the modeling error and the external disturbance; the simulation results demonstrated that the tracking error was reduced to 1% [19]. Liu L et al. proposed a compound control strategy using a feedforward-feedback structure and the Preisach model estimation was the output of the feedforward controller while the Preisach density function was achieved by using least squares estimation, and the proportional integral feedback controller was used to suppress disturbances for robustness [20]. Lechevin N et al. proposed a quasipassivity-based robust nonlinear control law that comprised a sliding mode control and Proportion Differentiation control that compensated for the delay induced by the hysteretic characteristics of the system. Simulations validated the proposed approach, illustrating that tracking of a sinusoidal trajectory led to a steady state error of less than 3%, which is acceptable for flap positioning [21]. Al Janaideh M et al. proposed a hybrid model that holds the hysteresis nonlinearity and the memory effects of the play operator, and simulation results showed the capability of the hybrid model to endow the hysteresis nonlinearity with memory effects [22]. Gu GY et al. proposed a hybrid control strategy combining a feedforward controller, which was an ellipse-based mathematic model, and a PID feedback loop for highly accurate and fast tracking control of piezoelectric actuators. The experimental results showed that the tracking performance was greatly improved and the root-mean-square tracking error was reduced to only 0.34% of the displacement range under a 100 Hz input frequency [23]. Nguyen BK et al. proposed a control strategy by combining feedforward controller using the fuzzy-based inverse Preisach model and feedback controller using the PID controller. The experimental results showed that the proposed control algorithm was available for the position control, and therefore the hysteresis effect of compensation of the SMA actuators is compensated [24]. Rosenbaum S et al. compared the hysteresis models from Jiles and Atherton and Preisach in their original scalar form with respect to their suitability for hysteresis-compensating control. The test results showed that the Preisach model worked smoothly within the control framework and performed more robust for the given task, and the implementation of the JCA model shows a certain sensitivity to the input increment [25].

We established a feedforward control to eliminate the negative effects of hysteresis nonlinearity. To achieve feedforward control of MSMA actuators, we used a hysteresis inverse model based on a Krasnosel'skii-Pokrovskii (KP) hysteresis model as a feedforward controller. To further improve the control precision and the performance of the adaptive system, we adopted a hybrid control scheme that respectively combines classical PID feedback control and self-tuning PID feedback control based on the Radial Basis Function (RBF) neural network, and implements high precision control of the output displacement of the MSMA actuator. Simulation results demonstrated the validity of the proposed approach.

## Methods

### Hysteresis Nonlinear Model of MSMA Actuator based on the KP Model

**Figure 1:**
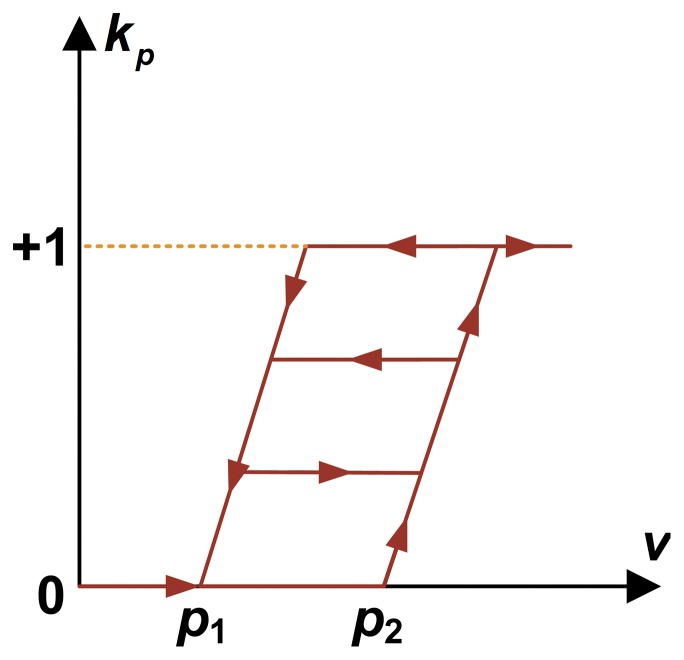
KP operator. The elementary hysteresis operator can describe the hysteresis nonlinearity with a minor loop. 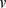 is the hysteresis input and 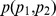 gives the output values of each KP operator.

**KP operator and discretization of the KP model.** For practical application of the actuator, the hysteresis loop curve of the actuator input-output relationship includes a major loop and a minor loop, and in this paper we establish a hysteresis non-linear model based on the KP model to provide an accurate description of the hysteresis loops in the MSMA actuator. The elementary hysteresis operator selected here is the KP operator (Figure 1) [26], [27]. To implement the KP model in a computer system, the corresponding Preisach plane is discretized. Assuming that each density parameter of the KP operator is 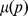, the discretized KP model formula is expressed as
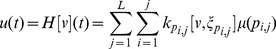
(1)


where 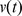 and 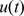 are the input and the output, respectively, of the KP model, 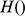 is the transformation operator between the input and output, 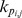 is the KP operator, 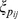 records the extreme output, the density parameter 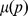 of the KP operator is utilized to weight the output of the operator, and 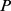 is the Preisach Plane, which can be expressed as
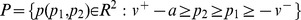
(2)


where 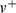 and 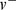 respectively are positive and negative hysteresis input extreme, 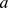 is the rise-constant of the KP operator, a pair of parameters 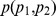 (see Figure 1) gives the output values of each KP operator,where



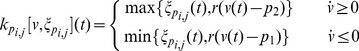
(3)the value of the memory variable 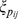 depends on the operator 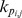 and is updated whenever the rate of 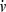 changes sign; the corresponding extreme function is indicated as
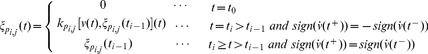
(4)



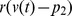
 and 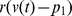 are the ridge function given by.
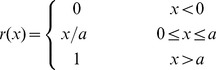
(5)


where 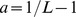, 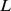 is the number of dividing lines, and above is the discretized KP formula.

#### Variable step-size recursive least-squares method

To obtain the appropriate density parameter, this paper employs the variable step-size recursive least-squares method, which reduces the amount of calculations by merging some repetitive operations in the traditional recursive least-squares algorithm [27], [28].

Suppose that the algorithm of the recursive weighted least squares is as follows:
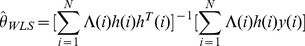
(6)


where 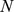 is the identified data length, 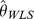 is the identified density function, 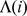 is a weighted factor, 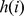 is the vector of the output of the KP operator, and 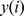 is the practical output value. Assuming 
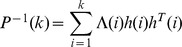
, 
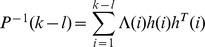
, 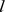 is the step-size parameter whose value is an integer greater than zero, let 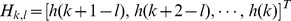, 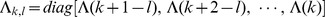, the recursion formula of variable step-size recursive least-squares can be described by:
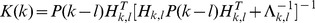
(7)




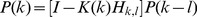
(8)




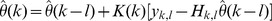
(9)


The step-size 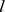 parameter stands for the revising weight as each 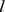 set of data is observed, and the speed and accuracy are adjusted by changing the value of 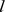. When using the variable step-size recursive least-squares method to identify the density function, the number of discretization lines is increased and the step-size 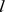 decreased, resulting in a more precise model of the hysteresis loop.

### Design of Feedforward Controller of the MSMA Actuator

**Figure 2:**
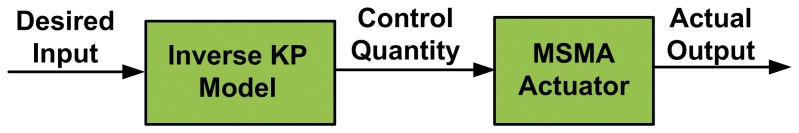
Feedforward control system for the MSMA actuator. The feedforward controller is an inverse KP model that can linearize the entire system by cascading MSMA actuators.

**Figure 3:**
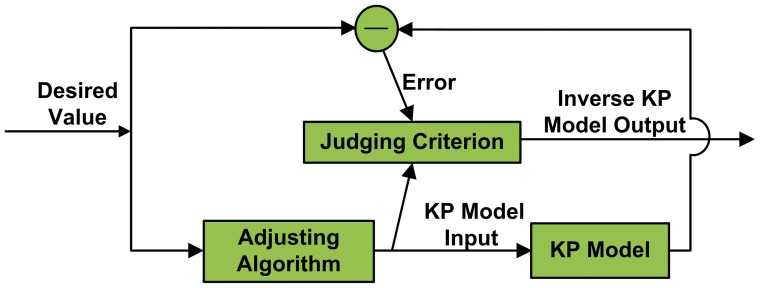
Solution method of the inverse KP model. First, provide the desired value to the KP model. Then compare the output with the desired value, input the resulting error into the judging criterion to adjust the algorithm until the output approaches the desired value.

To linearize the entire system, we established an inverse model as the feedforward controller for the MSMA actuator [24], [29]. The schematic of the feedforward control system of the MSMA actuator is shown in Figure 2. An explicit solution method is used to obtain the inverse of the KP model by adjusting the input of the KP model to explore the solution [30]. First, the input of the KP model is initialized and then as the input data are entered into the model, the calculated output data are compared with the desired output to obtain the error, which is used to vary the input until the output from the model approaches the desired output value. Now the explored input is regarded as the output of the inverse KP model and the schematic of the explicit solution method is shown in Figure 3. The judging criterion in Figure 3 means that the output error in the KP model is lower than the desired error and the adjusting algorithm is described as
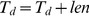
(10)


where 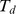 is the current input of the KP model, and 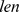 is a constant used to adjust the step-size. Here we propose a modified algorithm that mainly modifies the step-size 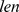: Set the initial value of 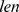 to 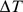 which is the width of the grid in the discrete Preisach plane, and in this way the error between 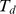 and the desired value will not be greater than 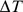. Suppose the error of the KP model is 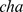, the step-size 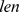 is expressed as
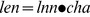
(11)


where 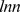 meets 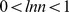.

**Figure 4:**
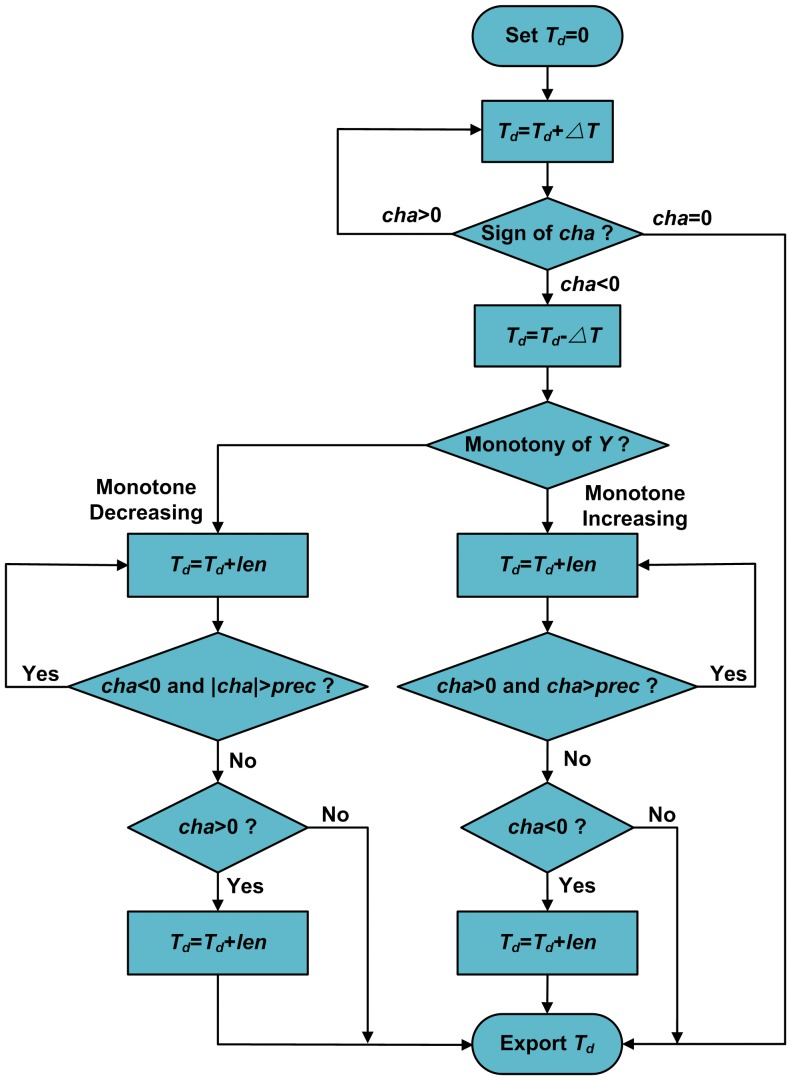
Modified solution method of the inverse KP model. 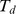
 is the input value of the KP model, initialize 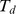 to be 0, 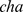 is the error of KP model, 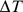 is the width of the grid in a discrete Preisach plane, 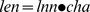 is the step-size. The inverse KP model is obtained by the adjustment the algorithm by judging the sign of 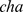 and the monotony of output 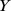. The error of the KP model 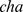 should be less than the permissible error of the KP model 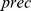.

The flowchart of the modified solution method of the inverse KP model is given in Figure 4, in which 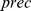 is the permissible error of the KP model.

### Hybrid Control Scheme of the MSMA Actuator

The previously proposed control scheme makes the inverse KP model a feedforward controller. To further improve the control accuracy and anti-disturbance performance, a hybrid control scheme is proposed by combining feedforward control and feedback control.

#### Hybrid control based on classical PID control

In this section, a control method is adopted by combining the inverse KP model feedforward controller with a feedback controller using the classical PID control method. The relationship between the output 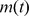 of the PID controller and the systematic deviation 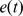 is described by
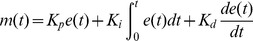
(12)


where 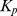 is the proportional coefficient, 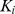 is the integral coefficient and 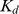 is the differential coefficient.

#### Hybrid control based on RBF neural network self-tuning PID

**Figure 5:**
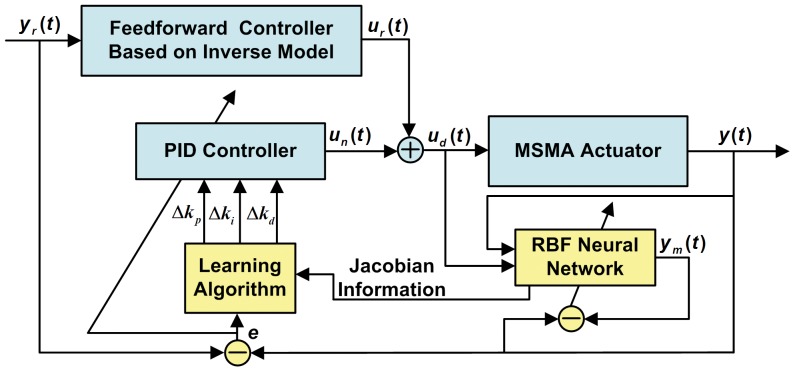
Hybrid control of the MSMA actuator based on RBF neural network self-tuning PID. In this system, 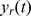 is the desired input, 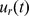 is the output of inverse KP model, 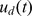, 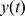, 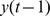 are the inputs of the RBF neural network, 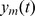 is the output of the RBF neural network, 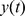 is the actual output, the Jacobian information is obtained from the RBF neural network and the parameters of the PID controller 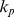, 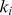, 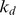, are adjusted via the gradient descent method, and 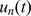 is the output of the PID controller.

In this section, classical PID control is exchanged for the RBF neural network self-tuning PID control [31]–[34] to further improve the control precision and adaptivity. The hybrid control structure for the MSMA actuator based on the RBF neural network self-tuning PID is illustrated in Figure 5.

**Figure 6:**
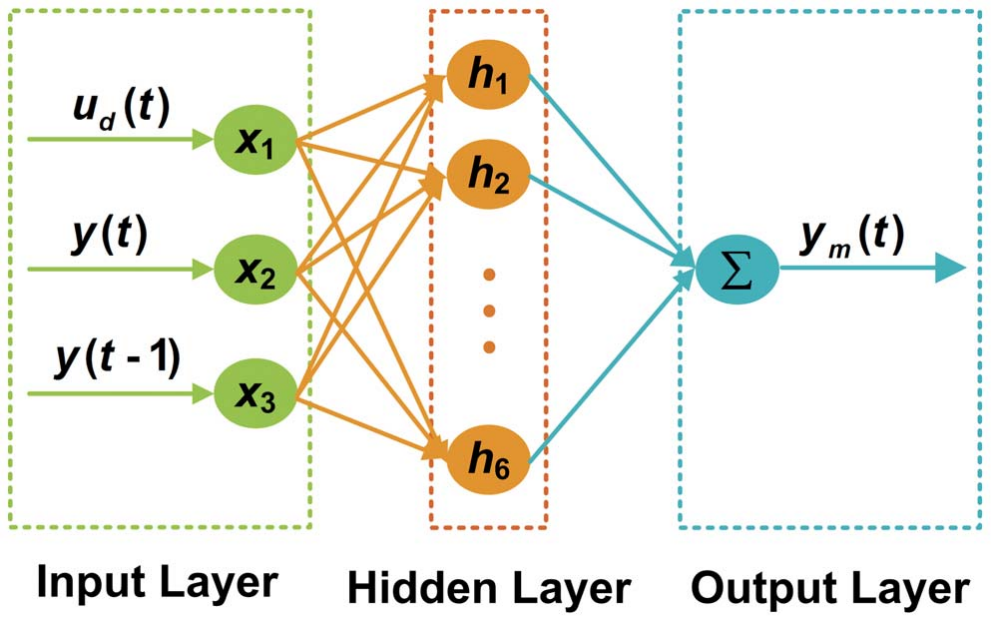
RBF neural network. The input layer has three neurons that represent the control signal at moment 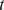, the output of the actuator at moment 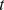, and 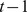; the hidden layer has six neurons; and the output layer has one neuron.

The RBF neural network is a three-layer forward neural network with a single hidden layer. The structure of the RBF neural network adopted in this paper is illustrated in Figure 6. The mapping from input to output is non-linear, while the mapping from the space of the hidden layer to the space of the output is linear. This structure not only ensures a faster learning speed but also avoids falling into a local minimum. As Figure 6 shows, the input layer comprises three neurons, which are the control signal of the actuator at moment 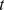, the output of the actuator at moment 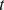 and the output of the actuator at moment 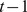. The hidden layer comprises six neurons and the corresponding RBF vector is expressed as
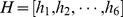
(13)


where the Gaussian basis function 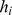 can be expressed as
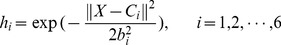
(14)


where 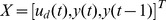 is the input vector of the RBF neural network, 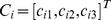 is the 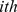 center vector 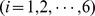, and 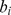 is the base width parameter of the 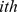 node.

The weight from the input layer to the hidden layer is taken as constant 1 and the weight vector from hidden layer to output layer is taken as 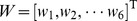 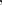 so the output of the RBF neural network is presented as:
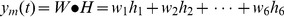
(15)


The learning performance function of the RBF neural network is chosen as:
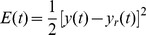
(16)


The gradient descent method is selected as the learning algorithm in the neural network.

#### Parameter-tuning algorithm of the RBF neutral network PID controller

The PID controller applied in this paper is an incremental PID controller and the control error is the difference between the input and output which is expressed as:
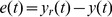
(17)


The tuning index of the tuning algorithm is defined as:
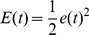
(18)


The parameters of proportion, integral and differential in the PID controller are 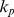, 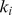, 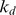 respectively, and the corresponding adjustment can be obtained as
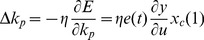
(19)

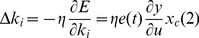
(20)

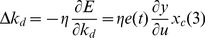
(21)


where 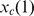, 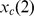 and 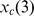 are the proportional, integral and differential respectively, and 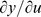 is the Jacobian information of the MSMA actuator which represents sensitivity of the output to the input of the MSMA actuator, so the Jacobian information is derived from:
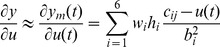
(22)


To sum up, the output of the PID controller can be expressed as:
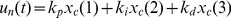
(23)


## Results

**Figure 7:**
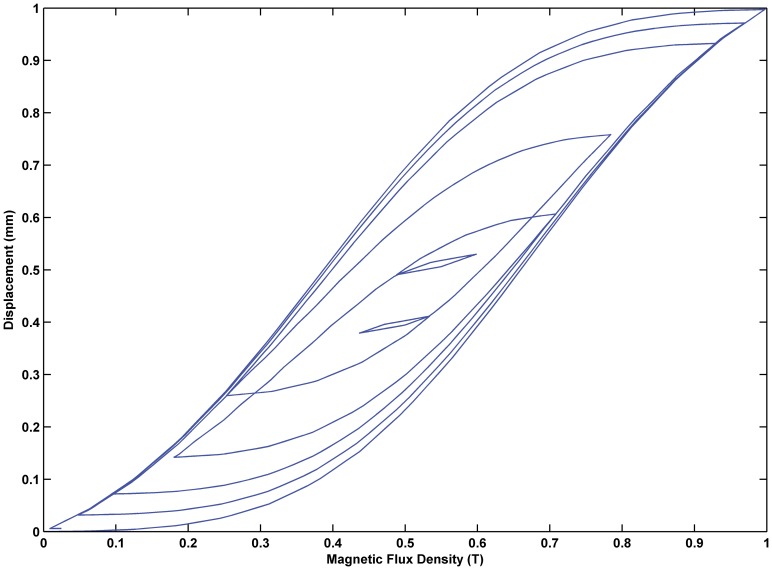
Relationship between input and output. The input is a magnetic flux density and the output is displacement, and there are major loops and minor loops in the hysteresis curve which can be applied to identification.

The KP model can be used to describe the hysteresis minor loop and demonstrate the complex input-output relationship. The relationship between the input and output for the MSMA actuators is shown in Figure 7. The hysteresis curve includes a major loop and a minor loop, which can be applied to the identification of the KP model. The input is magnetic flux density and the output is displacement.

### Simulation Experiment of Inverse KP Model for the MSMA Actuator

**Figure 8:**
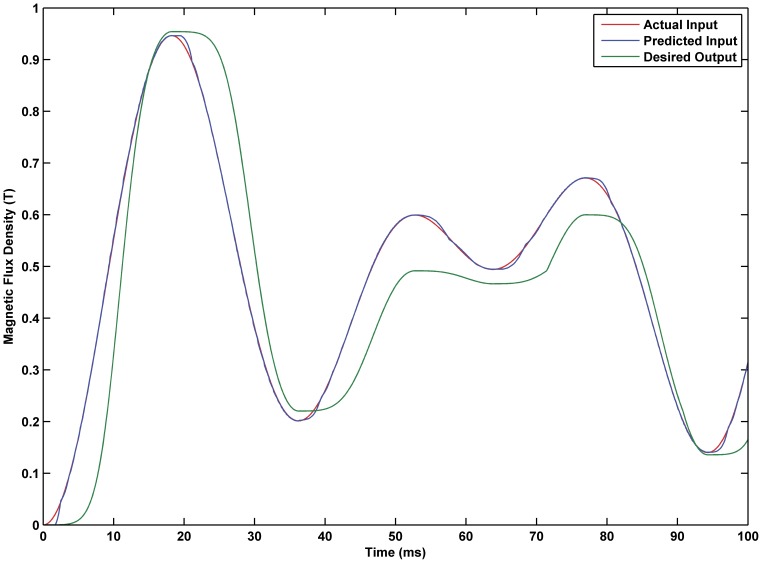
Predictive input of the inverse KP model. Comparison of predictive input and actual input of the inverse KP model.

**Figure 9:**
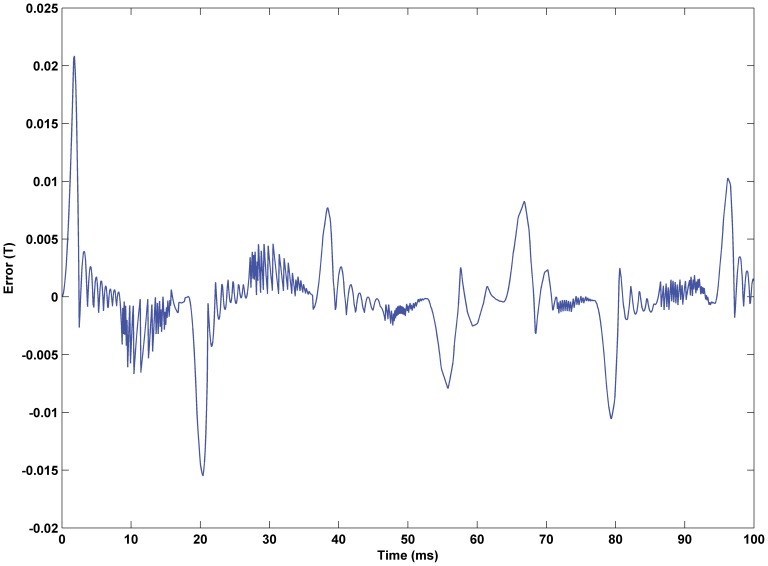
Error of predictive input for the inverse KP model. The maximum error of prediction is 2.09%.

**Figure 10:**
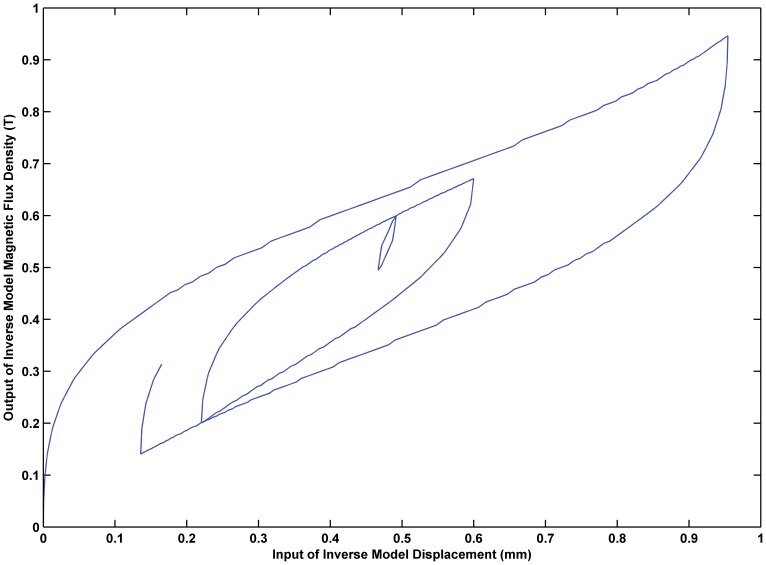
Input-output hysteresis loop of the inverse KP model. Input-output hysteresis curve of the established inverse KP model.

To verify the validity of the inverse KP model proposed in this paper, the number of discretization lines 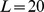. When the output data serve as the input data of the inverse model, and the parameter 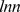 is 0.5, 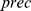 is 0.0001, the resulting simulation is shown in Figure 8–10. The simulation results show that the proposed hysteresis inverse model for the MSMA actuator is able to achieve a fine accuracy. When setting 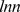 to 0.5 and 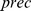 to 0.0001, the maximum error of the model is 2.09%.

### Simulation Experiment of Feedforward Control for the MSMA Actuator

**Figure 11:**
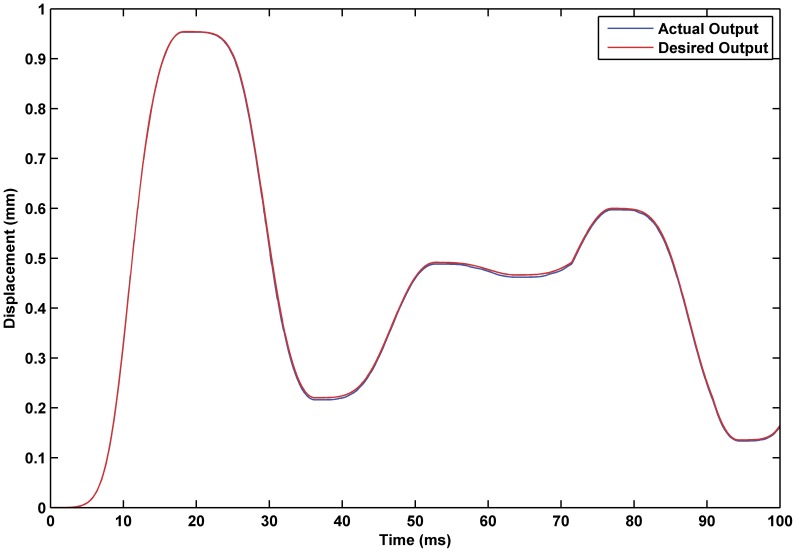
Displacement tracking based on feedforward control. Tracking effectiveness of the actual output and desired output under the feedforward control system.

**Figure 12:**
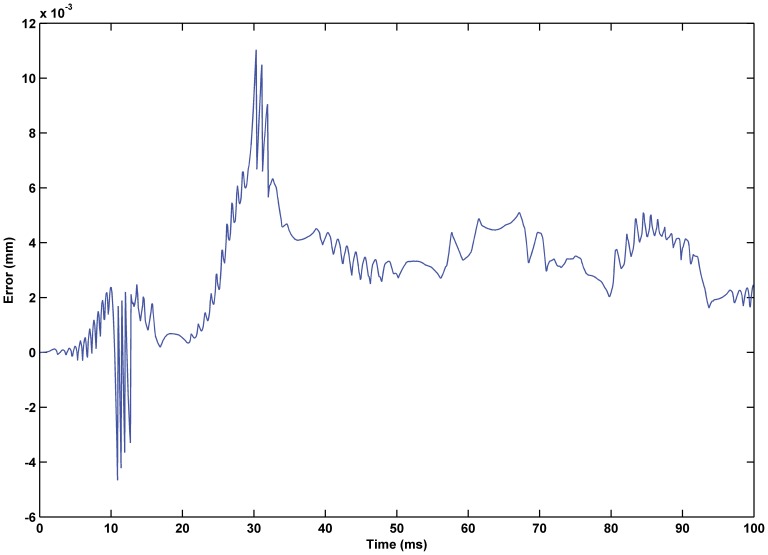
Error of displacement tracking based on feedforward control. The tracking error in the feedforward control system shows that the maximum tracking error is 1.1%.

In this feedforward control experiment, when we make the desired displacement an input signal, in an ideal situation, the input-output relationship should be linear. Figure 11 illustrates the effectiveness of the feedforward control with the parameter 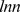 set to 0.3. Figure 11 shows that the entire feedforward control system tracks the control signal well and the maximum tracking error is only 1.1%. Figure 12 shows the tracking error for the whole feedforward control system.

**Figure 13:**
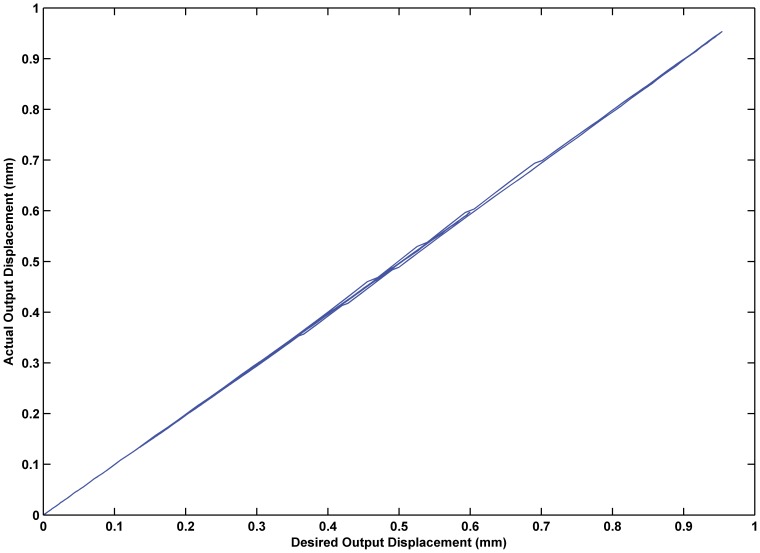
Input-output relationship in feedforward control system. Effectiveness of feedforward control, which makes the entire system input-output relationship linear and controllable.

The feedforward controller designed in this paper compensates for the hysteresis nonlinearity so that the input-output relationship can transform a complex uncontrollable hysteresis loop to a simple controllable linear relationship. Figure 13 demonstrates the effectiveness of feedforward control. According to the diagram, the feedforward controller established in this paper makes the entire system input-output relationship linear, which vastly enhances the controllability of the system. Therefore, the design of the feedforward controller achieved the desired goal.

### Simulation Experiment of Hybrid Control based on PID Feedback

**Figure 14:**
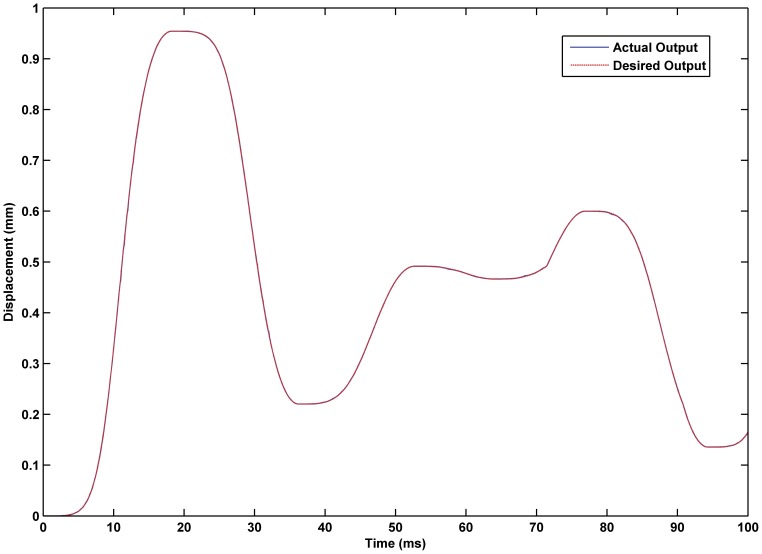
Displacement tracking based on PID feedback hybrid control. Tracking effectiveness of actual output and desired output under PID feedback hybrid control.

Based on a large number of experiments and parameter adjustments, the differential coefficient 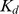 could be obtained as 0, the proportionality coefficient 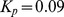, and the integral coefficient 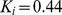; the resulting simulation is shown as Figure 14 through 16. The experiment results show that the PID feedback element results in an obvious improvement in the system control accuracy, and the tracking error was reduced from 1.1% in the open-loop system to 0.61%. In addition, the linear input-output relationship was further improved so that the system was more controllable.

**Figure 15:**
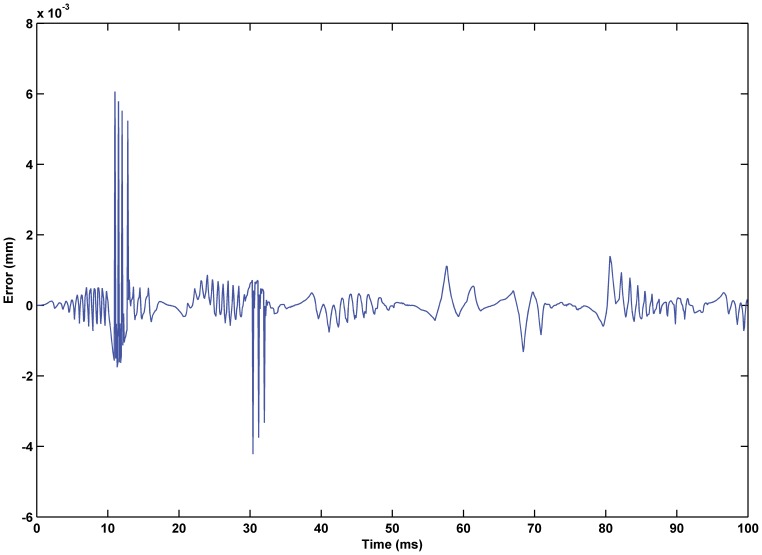
Error of displacement tracking based on PID feedback hybrid control. The tracking error was reduced from 1.1% in the feedforward control system to 0.61%.

**Figure 16:**
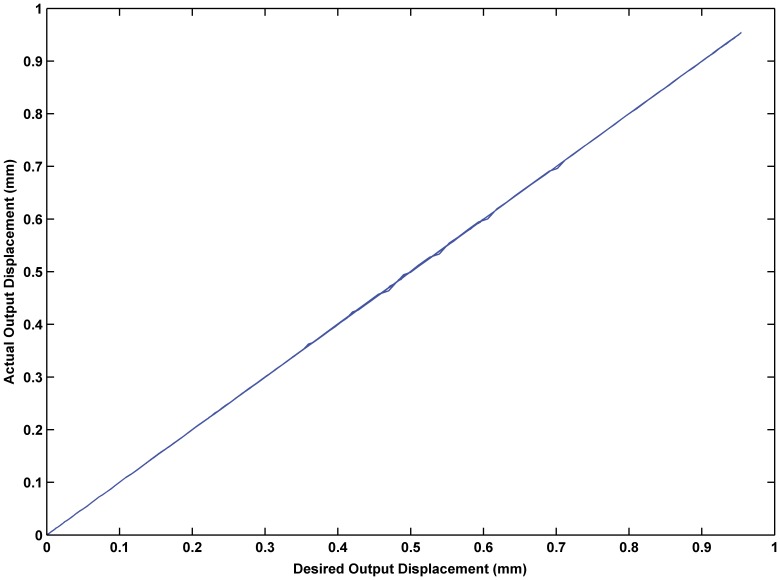
Input-output relationship based on PID feedback hybrid control. The PID feedback hybrid control is effective and the linear input- output relationship is improved compared with the feedforward control.

### Simulation Experiment of Hybrid Control based on the RBF Neural Network Self-tuning PID

**Figure 17:**
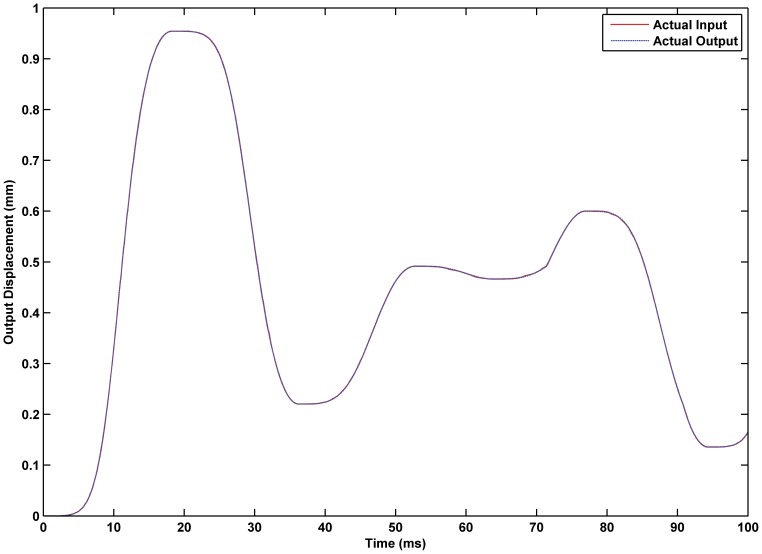
Displacement tracking based on RBF neural network self-tuning PID hybrid control. Tracking effectiveness of the actual output and desired output under RBF neural network self-tuning PID hybrid control.

To verify the effectiveness of the hybrid control scheme based on the RBF neural network self-tuning PID, we performed a simulation experiment. In this experiment, the parameters of the PID controller were set to 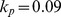, 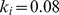, 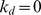, initial weight value was set to a random value ranging from 0 to 1, the learning rate was set to 0.6 and the initial value of 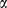 was 0.05, 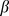 was 0.02. The experimental results are shown in Figure 17 through 19. The results of experiment based on the RBF neural network self-tuning PID hybrid control demonstrated that the system accuracy was improved and the maximum tracking error was reduced from 0.61% using a classical PID hybrid control to 0.43%. Figure 19 illustrates the RBF neural network self-tuning PID hybrid control affecting the entire system.

**Figure 18:**
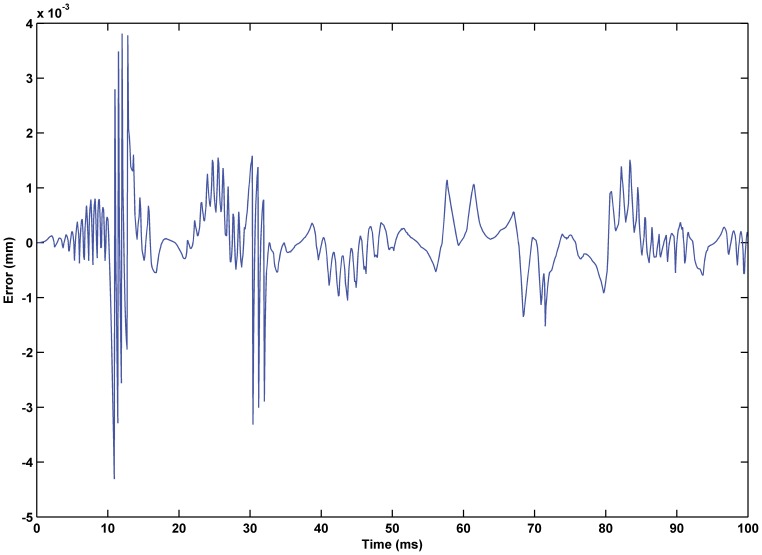
Error of displacement tracking based on RBF neural network self-tuning PID hybrid control. The tracking error was reduced from 0.61% using classical PID hybrid control to 0.43% using the RBF neural network self-tuning PID hybrid control.

**Figure 19:**
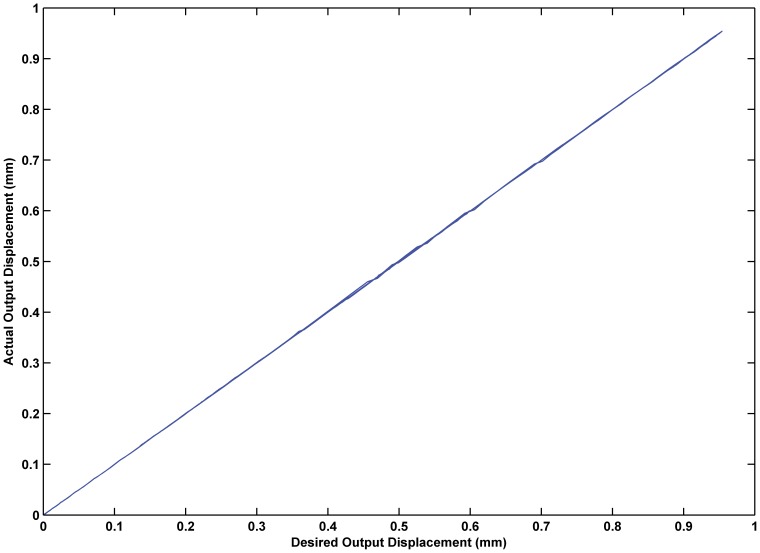
Input-output relationship based on the RBF neural network PID feedback hybrid control. The RBF neural network self-tuning PID hybrid control is effective and the linear input-output relationship is improved compared with PID feedback control.

## Discussion

This paper presents a hybrid control strategy for an MSMA actuator that utilizes the inverse KP model as a feedforward controller with PID feedback control. The proposed hybrid control strategy is applicable for all the type of MSMA actuator whose output displacement is continuous. We identified the density parameters in the KP model using a variable step-size recursive least-squares estimation algorithm. Based on the KP model, an inverse KP model was established by employing the modified explicit solution method, and the inverse model served as a feedforward controller to implement open-loop control for the MSMA actuator. To further improve the control accuracy, we combined PID feedback control and a feedforward control system, and then replaced the classical PID controller with a self-tuning PID controller based on the RBF neural network. The Jacobian information of the controlled object was obtained from the RBF neural network to adjust the PID parameters in real-time, enhancing the adaptive performance of the system. The simulation experiment results illustrated that the inverse model established in this paper possessed good predictive accuracy with a maximum prediction error of 2.09%. In addition, the entire feedforward control system could track the control signal well and the maximum tracking error was only 1.1%, while, when combined with the classical PID control, the maximum tracking error was reduced to 0.61%. Finally, the maximum tracking error decreased to 0.43% when the classical PID control was replaced by the self-tuning PID controller based on the RBF neural network. Therefore, the simulation experiment verified the validity of the hybrid control strategy.
